# Exploring drivers of food choice among PLHIV and their families in a peri-urban Dar es Salaam, Tanzania

**DOI:** 10.1186/s12889-022-13430-3

**Published:** 2022-05-30

**Authors:** Morgan Boncyk, Aloisia Shemdoe, Ramya Ambikapathi, Dominic Mosha, Savannah L. Froese, Cristiana K. Verissimo, Mary Mwanyika-Sando, Japhet Killewo, Germana H. Leyna, Nilupa S. Gunaratna, Crystal L. Patil

**Affiliations:** 1 Department of Public Health, Purdue University, West Lafayette, USA; 2 African Academy of Public Health, Dar es Salaam, Tanzania; 3 Tanzania Food and Nutrition Centre, Dar es Salaam, Tanzania; 4 Department of Nutrition Science, Purdue University, West Lafayette, USA; 5 Department of Epidemiology and Biostatistics, Muhimbili University of Health and Allied Sciences, Dar es Salaam, Tanzania; 6 Department of Human Development Nursing Science, University of Illinois Chicago, Chicago, USA

**Keywords:** Food environment, Family perspective, Food choice, Chronic disease

## Abstract

**Background:**

A nutritious diet is critical to minimizing disease progression of human immunodeficiency virus (HIV) and maximizing treatment efficacy. In low resource settings, meeting the food preference needs of people living with the HIV (PLHIV) can be achieved with a supportive food environment when HIV status is disclosed. However, less is known about family-level strategies related to building a supportive food environment. The Diet, Environment, and Choices of positive living (DECIDE), a mixed-methods observational study conducted in peri-urban Dar es Salaam, Tanzania, explored food preferences as influenced by the personal, family, and external food domains.

**Methods:**

We completed a qualitative analysis of data generated from 40 interviews (*n* = 20 PLHIV and *n* = 20 family members) aimed at exploring the dynamics of food choice for using a family perspective. We expanded on Turner’s food environment framework and drew on Giddens’ structuration theory to guide our data collection and analysis. Interviews were audio recorded, transcribed, translated from Kiswahili to English, coded, and organized into themes.

**Results:**

We found PLHIV personal food preferences were influenced by organoleptic properties, medications, disease stage, and gender norms. Family members were knowledgeable about the importance of nutritious food for HIV treatment and prioritized these needs to avoid HIV-related stigma and fulfill family obligations. With high prices of nutritious foods (animal source foods, fruits), family members strategized to secure preferred foods for the PLHIV by, 1) forgoing their own food preferences; 2) reallocating food within the household; 3)making food substitutions; and 4) leveraging external networks. These strategies were increasingly employed as the disease progressed.

**Conclusion:**

The use of this expanded framework that included a family perspective on PLHIV food choice illuminated the various households decision-making dynamics that took place in this low resource community. Family members of PLHIV tried to buffer the limitations imposed by the external food environment, especially as the disease progressed. In the context of HIV status disclosure, integrating a family perspective into HIV nutrition interventions and programs has the potential to influence health outcomes and slow disease progression.

## Background

Approximately 38 million people globally are infected with the human immunodeficiency virus (HIV), and about 1.6 million people with HIV reside in Tanzania [1]. Because diet is critical to minimizing disease progression of HIV and maximizing treatment efficacy, nutrition and food security play a central role in health outcomes of people living with HIV (PLHIV) [2]. Historically, alleviating aspects of household food security has positively affected medication adherence [3]. Several studies report that physical disease symptoms and overall disease progression relate to food preferences of PLHIV [2, 4–8]. Caregivers in Lesotho recognized that food intake could slow HIV disease progression and that the appetite of a PLHIV continuously reduced with increasing severity [2]. Family perspectives on medication adherence have been well documented [2, 9–15], while very few studies have examined family’s role with PLHIV diet and food choice. As HIV is managed over the long term and PLHIV age, common co-morbidities (tuberculosis, opportunistic infections) and a newer syndemic of diet-related non-communicable diseases (hypertension, diabetes, metabolic diseases) become relevant, further affecting the dietary needs of PLHIV [16, 17]. Food choice, the process through which humans make decisions on what and why they eat those foods provides useful social and physical context that are insightful for interventions and policies [18]. However, fewer studies include perspectives of other household members to better understand the drivers of food choice among PLHIV. Understanding the drivers of food choice among PLHIV families can provide important context for interventions.

To understand the family context of drivers of food choice among PLHIV families, we use a food environment framework and Giddens’ structuration theory [19, 20]. Food environment, as framed by Turner et al. [20], influences food choice through two domains: external (i.e., food availability, pricing, vendor and product properties, marketing) and personal (i.e., food accessibility, affordability, convenience, desirability) [20]. In low-income settings, there is considerable evidence on external domains while very few studies focus on the interaction between personal and external domains [20]. Complementary to the food environment framework is the Giddens’ structuration theory which explains how social structures influence agency (“food choice”) to create routines that can change over time [19]. Here, food choice is influenced by cultural [5, 6] and structural drivers (ecological, economic, and social) [21]. These structures and food choice duality to influence household decisions about food and food allocation [3, 22]. In limited resource settings, PLHIV and their caregivers face unique challenges, while living with and managing a chronic illness [21, 23]. Here, we examine the food environment and identify and explore the role of family (social structure) in dynamics of food choice for PLHIV who had disclosed their HIV status to family members in peri-urban Dar es Salaam, Tanzania [20, 24].

## Methods

### Study overview

The qualitative data analyzed for this paper was collected as part of the “Diet, Environment, and Choices of positive living: Evaluating personal and external food environment influences on diets among PLHIV and families in Dar es Salaam, Tanzania study” (DECIDE). DECIDE study used multiple methods with qualitative, quantitative and geo-spatial data collection to evaluate external and internal food environments in peri-urban Dar es Salaam of PLHIV families [25]. It is nested within the ongoing Dar es Salaam Urban Cohort surveillance (DUCS), where 21,000 households are routinely assessed for vital events, migration, and other demographics [26].

Relevant guidelines for the DECIDE study were carried out in accordance with the Institutional Review Boards at Purdue University (USA, #1806020735) and the National Institute of Medical Research (Tanzania, #2899). All participants went through the informed consent process. To ensure confidentiality and anonymity, all potential identifiers were replaced with pseudonyms.

### Procedures

PLHIV who had disclosed their HIV status and family members were recruited from two HIV clinics, one associated with a large hospital and a second is a dispensary around the DUCS area when they attended or accompanied a PLHIV for a health visit. PLHIV with knowledge of their HIV status and lived within or near the DUCS study area were eligible for interviews. Participants were excluded if they were under the age of 18 or unable to communicate in Swahili. Recruited PLHIV then identified family members they had disclosed their status to, influenced food preparation, and provided consent for researcher to contact them for participation in interviews. Interviewed PLHIV and family members were not from the same household, so the sample did not consist of dyads. Participant recruitment aims to provide a diverse set of male and female caregivers for male and female PLHIV. All participants provided consent to participate and met at a convenient time and location for the interview. Men and women were interviewed as separate populations to explore genders influence on food and food choices. Maximum variation technique [27–29] was used to recruit participants for age, gender (female-headed household) of PLHIV, food security status, HIV disclosure, time on ARVs and other co-morbidities, BMI categories, and socio-economic status.

A theory-informed thematic analysis was used to describe individual food experiences emphasizing their own perspectives [30, 31]. Face-to-face semi-structured interviews (60–90 min) were conducted in Kiswahili by an experienced Tanzanian qualitative researcher (AS) between December 2018 and May 2019. The interviewer also used reflective notes to capture initial impressions and track emerging codes and themes. Recordings were transcribed verbatim and checked for accuracy by the research team. Swahili transcriptions were translated to English and read side-by-side to check for translation and aid interpretation.

The interview guides and analytical codebook were informed by Giddens' structuration theory [21, 23] and Turner's food environment framework [20] to allow us to explore core motivations and perceptions of food environments and how these affect PLHIV household food choice and intra-household allocation of food and dietary intake. Questions specifically asked about PLHIV food choice, changes since diagnosis, food procurement and processing, water security, home gardening, and nutrition knowledge. The family members’ interview guide mirrored the PLHIV’s guide structure, except that it referred to PLHIV’s food choice. Interview guides were pretested and revised three times before data collection (Table 1).

**Table 1:** English translation of the semi-structured interview guide

**CHARACTERISTICS OF FOOD**
Which food smells do you enjoy?
Which food smells do you dislike? Which smells bother you?
Which flavors are attractive to you? Sweet like sugar? Salty? Sour like lemon? Bitter like African eggplant?
Which flavors to you avoid?
Which food feelings, textures, or sensations do you like?
Which food feelings, textures, or sensations do you dislike or avoid?
Which foods make you feel full and satisfied? In other words, which foods provide you with comfort?
FOR HIV+ only: How has your disease/diagnosis affected what you eat? Are there any patterns to how these have shifted over time (since diagnosis, with different treatment options)?
**FOOD ENVIRONMENT AND THE HOUSEHOLD**
Sometimes people in the same household have different food wants and needs. Talk to me about this
Do others in your home affect your food choices? Does everyone eat the same (including ones who are sick or children? How does this affect your time (spent shopping, cooking) and resources (food cost)? Do additional visitors bring you a financial cost?
**HEALTH AND FOOD**
Are there foods that you cannot eat?
Are there foods that you crave or prefer to eat?
Are there foods that you want to eat, but that you are not eating?
When you are sick, how does your diet change? Are there only certain foods you can eat? Does your medications affect what you eat?
**BUYING FOOD**
On a typical day, roughly how much do you think you spend on food?
What challenges do you have in your household with buying food? What causes these challenges?
Have you been affected by economic changes? Can you describe what has been happening and how it has affected the foods you eat, buy and grow?
What strategies do you use to overcome these challenges? Does anyone help you when you do not have enough money? What do you do when you do not have enough money?
**MEANING OF FOOD**
When I say, "healthy food", what does this mean to you?
When I say, "food is culture", what does this mean to you?
When I say, "food brings people together", what does this mean to you?

Rigor was maintained throughout the data collection and analysis by giving careful attention to interview quality through constant comparison of the aims for each interview question, using multiple data coders, reflective notes after each interview, an audit trail of all research activities, and observations discussed at team meetings [29, 32, 33].

### Analysis

A total of 40 participants were interviewed: 20 PLHIV and 20 family members (Table 2). English transcriptions were imported into MAXQDA, an application for managing, analyzing, and presenting qualitative research data [34]. Demographic and health data from the participants were also collected and described. The study team read the transcripts multiple times; reviewed field notes and ideas for initial coding, which were completed after each reading and iteratively edited after reading each interview [35]. Each transcript was coded by a primary (MB) or secondary (CV, RA, SF) coder, and findings were confirmed by six authors (AS, CP, CV, MB, RA, SF). From initial codes, the team organized the data into themes [36].

**Table 2:** Demographics of DECIDE qualitative study participants (n = 40) in peri-urban Dar es Salaam, Tanzania^a^

Qualitative Demographics	PLHIV (*n* = 20)	Family Member (*n* = 20)
**Sex**	12 Female	14 Female
**Age**^b^ **(years)**	38 (30.8, 45.8)	39.5 (28.3, 44.8)
**Education**^b^ **(years)**	7 (7, 11)	7 (7, 7)
**Marital Status**	6 Married	12 Married
**Relationship to PLHIV**^c^	-	15 Immediate family
**HIV + **	20	4

^a^Study participants were recruited from community-based HIV clinics at a hospital and dispensary; ^b^Information is reported as median (25% quartile, 75% quartile); ^c^Immediate family member indicates significant other, parents, siblings, and children

## Results

Three major patterns of meaning were identified from these data. The first theme captures the drivers of food choice for PLHIV. Second and third themes reflect family dynamics and what family members do to support food preferences and choices. Second theme shows how the family is motivated to support the food choices of PLHIV. Lastly, third theme explores the various strategies that are used to navigate constraints while trying to meet dietary needs and preferences of the PLHIV. The themes have been summarized into Table 3.

**Table 3:** Emerging themes organized by food environment domains with example behaviors

FOOD ENVIRONMENT DOMAIN	EMERGING THEME
**EXTERNAL**	**Food prices:** There were constant price fluctuations of food that were the primary driver of food purchase decisions.
	**Food vendors:** Purchase locations provided PLHIV and their families food on credit to pay back later, when they found work, so they would avoid going without eating.
	**Food availability:** It was difficult to plan food purchases in advance as the foods that were available for purchase differed by day.
**HOUSEHOLD/ FAMILY**	**Gender**: Women caregivers would forgo their own food needs and preferences to prioritize the needs of the PLHIV.
	**Disclosure and knowledge:** Within this disclosed population, family members knew that PLHIV needed to take their ARV medications with food to reduce side effects and improve the effectiveness of the medication.
	**Allocation decisions**: were made within the family to improve the diet of the PLHIV. • Especially with increased disease severity, family members would forgo their preferences to increase the food consumption and dietary quality of the PLHIV. • Cooking resources (one pot, fuel, water), time use, and affordability limited overall family food choice. Despite these constraints, PLHIV were often given a bigger portions and more desired foods. • Nutritious foods recommended by healthcare professionals were purchased for the PLHIV. • Families made food substitutions to reduce expenditure wherever possible.
	**Extended networks** helped to reduce resource insecurities and ameliorate extreme economic hardships. • Drawing on kin • Buying food with loans or on credit
**PERSONAL**	**Desirability**: • Preferred foods were chosen based on emotional connections (traditional, cultural, tribally relevant foods), specific tastes and smells but less on texture and appearance. • Change in food preferences over time with diagnosis and disease progression. • Gender differences exist in the perception of “healthy” foods.

### Theme one: Drivers of food choice among PLHIV

Only a few participants did not identify food preferences affecting caregivers’ or PLHIVs’ diet choice. As one young 22-year-old male caregiver with a mother who is HIV+ said, *“I never noticed that she dislikes any food, she eats all foods.”* Similarly, an older mother, aged 56, caring for her daughter said, *“She likes all foods … She does not choose.”* In parallel, some PLHIV did not identify any food preferences. A 33-year-old woman stated, *“there is no food that I can’t eat.”* Few PLHIV did prefer cooking for themselves. A 24-year-old male PLHIV explained, *"I love biriani because I can cook it."*

Within the personal domain, PLHIV preferences were influenced by gender and culture as well as the organoleptic characteristics of food (i.e., smell, taste, texture, appearance) and feelings of satiety. While on ARV treatment, personal food choices are shaped by changes in medications, severity of the disease, and the presence of other illnesses, such as TB. Together these factors contributed to one’s emotional connection with food and ultimately drove PLHIV’s food choice and diet.

#### Gender and culture

When asked about which foods made the PLHIV feel full and satisfied, men often described feelings of comfort and satiety from stiff porridge. As an HIV+ male simply stated, *“I am satisfied with stiff porridge.”* Women mentioned a wider variety of foods, including meats, seafood, vegetables, and stiff porridge. Many PLHIV reported that tradition and linkages to religion or one’s ethnicity (i.e., tribe) explained why certain foods were consumed, such as meat, plantains, and stiff porridge. A 33-year-old female with HIV explained, *“I don't know for other tribes [laughter] but mostly, every tribe has its own food that they prefer.”* Another woman, age 38, similarly stated that it, *“depends on one's origin for example, people from Arusha prefer plantain soup, that is their culture. I stayed at Arusha, but I am a Nyamwezi. Nyamwezi and Sukuma like stiff porridge so much.”*

#### Organoleptic properties

When asked about preferences and aversions, PLHIV often mentioned taste as the main reason for avoiding certain foods; however, there was no universal consensus on avoiding specific tastes. Several participants linked taste to body reactions. A 46-year-old female who has been diagnosed with HIV for 11 years stated, *"…when I eat lime, [it] makes the blood in my ears run cold."* A 39-year-old female caregiver for her HIV+ aunt reported, *"when she [PLHIV] eats sour foods, she feels her stomach is scratchy."* Sweet and sugary beverages, such as soda and juice, were mentioned as the cause of nausea by some; so, these beverages were avoided. Others preferred sweet and sugary beverages. One twenty-four-year-old male stated: *"two days cannot pass without drinking soda."* Often foods were avoided by some and desired or preferred by others. A female family caregiver in her late 20 s indicated that her HIV+ mother disliked sour tastes. In contrast, a 45-year-old female who got her HIV diagnosis nine years ago felt that the sour taste was good for her. She said, “*I like sour taste; it brings food appetite.”*

Other organoleptic properties, such as texture and appearance, were less frequently mentioned. Appearance was often used to compare and differentiate foods such as meat and seafood. Several participants did talk about disliking the texture of certain fruits and vegetables (e.g., watermelon, jackfruit, cabbage) and relishes. When PLHIV mentioned a dislike for certain textures or appearances, they connected this to decreased appetite. For instance, a male PLHIV in his mid-twenties mentioned *“I don’t like foods which are cooked with lots of soup, I don’t like its appearance. When I see them, my appetite vanishes.”*

PLHIV connected preferred foods, tastes and smells to emotions, satiety, and occasionally spirituality. A 33-year-old man diagnosed nine years ago stated: *"if I eat cooked plantains, I am satisfied … my soul is completely satisfied."* Another male in his early forties who was diagnosed two years ago said, *"There is one thing that God has given me. If stiff porridge is being cooked at home when I smell it, I feel happy. But if it is from a food vendor, I will know that today I cannot eat that stiff porridge at the food vendor place. I can't eat it."*

#### Duration of ARV treatment, HIV severity, and co-morbidities

PLHIV who had been on ARVs for a longer duration recognized that the medication changed their food preferences, others were unsure what caused these changes to occur. Women on ARVs for a longer duration were in tune with foods to avoid. A female in her mid-forties diagnosed 11 years ago said: *"When I eat hot rice, I do not get full. I prefer cold rice."* Another 38-year-old woman diagnosed 10 years ago said:

*"The flavor I liked was a sweet smell like the sugary taste, but I feel like in these recent weeks it does not fit me. For example, I used to love Mirinda soda, but nowadays when I drink it, its flavor remains here, and it really hurts me. I still love its smell, but it is not like how I used to love it previously. Those days when I walk, and it is sunny as I arrive at a place, I drink cold water then I drink Mirinda soda."*

One 24-year-old PLHIV, said it was difficult for him to know whether his HIV status, the ARVs, or treatments for other co-morbidities were complicating his food preferences.

*“This treatment has changed my food taste, but I can’t speak much about this because it has only [been a] few days since I have started using the [ARV] medicine. My appetite was reduced because I have been using two types of medicines for UTI and AIDS.”*

### Theme two: Family motivation

Among PLHIV that have disclosed to at least one family member, we identified several motivational factors driving family members support for PLHIV food choice and prioritization of their food consumption. First, knowledge about the relationship between health and diet was important.

#### Family nutrition knowledge

When asked about the foods that they define as healthy, men and women did respond differently. Women categorized meat and rice as unhealthy with vegetables, grains, fruits, beans, and eggs as healthier. Men deemed meats to be healthy, along with grains, fish, and fruits. Both men and women defined a balanced diet as one that included proteins and vitamins. Women positively stated carbohydrates and a dietary diversity of food to be healthy. In their descriptions of a balanced diet, men included fat, minerals, and satiety. Many PLHIV participants noted that a diverse or balanced diet was important for living with this disease. A 30-year-old PLHIV defined healthy foods as *“Vegetables, fruits, raw maize, stiff porridge from unhusked, maize, local eggs, and not broiler chicken… I mean eating different kinds of food.”* Occasionally, there were disagreements among family members about what should be eaten. A woman in her early thirties who was diagnosed eight years ago explained: *“I like meat, but my aunt always tells me to minimize the intake of meat since it is not good.”*

Knowledge about nutrition was high among participants because the PLHIV in this study disclosed their status to their family members; their family was involved in their healthcare. Both the PLHIV and their family members received health promotion messages and advice at health facilities about the importance of good nutrition; this influenced food choice decisions. PLHIV stated that they followed recommendations from healthcare providers who emphasized eating high nutritional value foods such as sweet potato leaves, cassava leaves, and other vegetables during more severe disease states. For instance, a 45-year-old HIV+ mother caring for her HIV+ daughter said, *“I really try hard… sweet potato leaves are always available in my house because of the sick person. She has been instructed by a doctor to eat sweet potato leaves every day.”* Many also knew that ARVs needed to be taken with food to work optimally. With this knowledge, family members worked to ensure that PLHIV consumed meals regularly for ARV efficacy. Some family said that they watched the PLHIV take their medication to ensure adherence. Others would encourage eating, and some would scold the PLHIV if they did not eat enough. For instance, a man in his early thirties and caring for his HIV+ younger brother said that we consider his “*condition, but we improve the food and make it good. So, he has his own food which is different from the family food … and [we] make sure he does not delay eating. He eats food at a proper time.”* Similarly, a 39-year-old female family member caring for her sister explained how the food choices of the PLHIV overrule those from other family members:

*“I do not give them [other family members a] chance [to make food requests] because if I give them, … my [HIV*+*] patient will not eat. I want to press those who are well [to forgo their food choices] for my patient to get [the] food[s they prefer] … Because if she is doing well, she goes up, you also get [a] relief. You can even get an emergency and travel while you know my patient takes her medicine. She is capable of sitting in the kitchen, she cooks, she eats. It makes my life easier.”*

#### Stigma and diet

PLHIV and their family members emphasized the importance of food to avoid appearing diseased. A 29-year-old woman mentioned that *“…no one knew if I was sick—that was because of food I was eating. The doctor told me I should eat lots of fruits together with starch food, not to eat chips. You see, it helped me a lot. Now I am trying to follow his/her directives.”* This avoidance of stigma also drove family members decisions. They prioritized PLHIV food choices to prevent the PLHIV from looking “diseased,” given the stigma associated with HIV and the belief that losing weight indicated HIV infection. A male caregiver, aged 32, said that *“He needs to eat, [and] drink lots of water”* to avoid looking tired and allowing others to judge him based on his disease status.

#### Family obligations

Family obligation was commonly cited as a reason for prioritizing PLHIV food consumption. While acknowledging the additional economic burdens, family caregivers justified this as part of their obligation to family. For example, a daughter viewed caring and making food decisions for her HIV+ mother in this way. She said, *“She is my parent. I have to make her satisfied. Even if I kept my money, I would spend it for her. She kept me to this age, I have to take care of her. This is the moment for her to be taken care [of] and I will get the blessings. So, I am ready to give her what she wants.”*

**Fig. 1:**
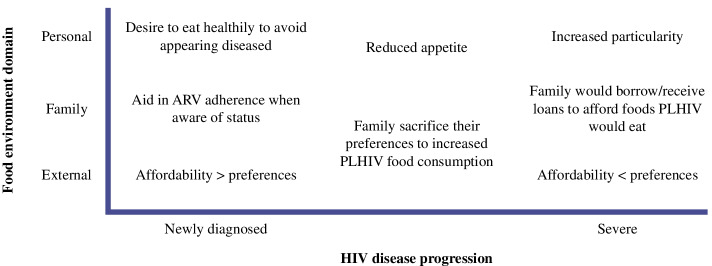
Drivers of food choice by time since diagnosis within each food environment domain

These obligations were especially apparent in times of severe illness. Family members noted that PLHIV food preferences became more specific with ARVs use and increased severity of illness. This often led to consuming foods that differed from what other family members ate (Fig. 1). In fact, family caregivers would allocate a larger proportion of the household’s daily budget to meet the special needs of the PLHIV. In other words, they would prioritize preferences over affordability. A 45-year-old daughter who was caring for her HIV+ mother mentioned how this was a family sacrifice: *"I am ready to give her what she wants. I may tell my young sister, ‘Today, I am stuck, please tell your husband to give me tshs 1000/* = *or 2000/* = *to assist our mother today she doesn’t want a certain food.’ We are trying to make sure we do what our mother wants.”* There were also changes in PLHIV food preferences over time as medication changed.

### Theme three: Family strategies

Most family members were able to describe the PLHIV’s food preferences. Family members discussed the various strategies that they used as they balanced economic challenges and their desire to ensure that nutritional needs of the PLHIV were met. Several family members described forgoing their own food choices or eating less so that the PLHIV could eat more desired foods. Others discussed the importance of relying on credit to access foods for the PLHIV. These intrahousehold money and food allocation changes varied based on the family’s access to foods and their nutrition knowledge, support, stigma, and feelings of obligation. While knowledge about nutritional needs was high, affordability played a large role in food choice. For instance, a recently diagnosed 50-year-old man said that a balanced diet will include fruits and food from other food groups, but that this knowledge did not influence his food consumption because *“… we eat food just for the sake of eating. but there is food to build our bodies, and other functions. It’s true those are [what we should eat], but for normal people like us it’s hard to reach that budget*.”

Food choices are narrowed by affordability and these financial forces impact the family's ability to meet PLHIV’s food choices. A 38-year-old man diagnosed with HIV for four years talked about the cost of living. He said, *"Life, the cost has risen. The value of money is the same, but life cost has risen."* Similarly, another man in his early thirties caring for his younger brother with HIV stated: *"food prices are high, and the value of money is somehow low."* A 43-year-old woman similarly explained, *"There is a challenge. There are other days I cannot afford [food] because I do not have [the] money, I hope you know that. This life and business are hard."* Unpredictable price fluctuations and uncertainty were stressors for PLHIV and the family member’s trying to support them. Given these economic constraints and the increased price of cooking separate meals, when the PLHIV were considered healthy, or their disease being managed, they would consume the same diet as the family. Meals were made within a given family budget. As a 39-year-old woman caring for her sister explained, *“We cannot afford to choose; you can’t make everyone happy.”* However, when their health worsened the PLHIV was given more desired parts of the food or increased quantities to ensure their medications would be effective.

#### Household food (re)allocation

In the context of financial constraint, people described substituting the desired food with something else to buffer the effects of changing food prices (Table 4). PLHIV commonly names foods they could not afford and what they chose to purchase instead. Generally, beans and vegetables were substituted for meats, while fish, chicken, and beef were bought when the household had money. Soda was consumed because it was cheaper than juice and milk. Porridge is a standard breakfast when unable to afford tea and snacks such as African donuts, chapati, or eggs. A greater economic constraint led to both substitution and reduced quantity. A man in his mid-forties (45 years) who had recently been diagnosed within the last year reported reduced bean consumption in addition to substitution of meat with beans. *“There are foods that I prefer, but I cannot afford to buy them, foods like watermelon, avocado, fish; I really like them."* The participants mentioned other financially inaccessible foods included eggs, French fries, and tea. Special foods, such as festival foods, are not consumed due to financial constraints.

**Table 4:** Food substitutions based on qualitative evidence

Representative Quotes	Preference	Substitution
*“I like juice, but it is unrealistic to buy and make juice, so I just drink soda. I prefer Fanta-Pineapple soda. If I could afford [it], I could drink soda every day, but because I cannot afford [juice], I rare[ly] drink [juice and instead drink] soda [laughter].”* (female, 45 years, 11 years since diagnosis)	Fruit juice	Soda
“*Yeah, it depends if the festival comes [around and] if we have money we eat [festival foods]. Other times we just cook normal food. We [will eat what we] usually eat if such specific food is not available.”* (female, 33 years, < 1 year since diagnosis)	Festival foods (e.g., spiced rice, meat dish)	Typical daily food
*“[In the] morning, if we do not have money, we make porridge and drink [it]. If there is money, we make tea with snacks, like wheat buns or chapati, we eat [that].” *(female, 39 years, sister is HIV+)	Snacks (e.g., wheat buns, chapati)	Porridge
*“In the afternoon, you can buy food for [a] side dish, [but] this is challenging because you can spend [up to] Tshs [Tanzanian shillings] 7000/ = . It[s price] depends [on what] you buy, [for example a] quarter kilogram of meat and its viungo [onions, tomatoes, okra, African eggplant, etc. [However], in [the] case I cook beans to eat with rice, the budget can be lowered.” *(female, 39 years, a stepchild is HIV+)	Meat with a vegetable side dish	Beans

Another strategy reported by family members was that they would forego their own food desires or consume less food to support and enable the PLHIV’s to consume their preferred foods. When a PLHIV had a strong food preference, several household members would sacrifice their own choices and accept the extra economic burdens required to accommodate this. For example, a 39-year-old female caregiver caring for her HIV+ mother said, “*She can't [be] denied food. She can say in the evening, she tells you: I wish I could eat kongoro [cooked animal leg], go and buy kongoro for me, I will eat with chapati, then I will sleep."*

Participants described coping with extreme food insecurity by skipping meals, most often the evening meal. A 29-year-old woman described her family food situation this way, she said, *"There is a challenge in a family. I might want to eat a certain meal, but I do not have it, that's a challenge. I have to accept that situation as a challenge."* When meals were skipped, PLHIV and family members will go to bed hungry, as a 57-year-old woman with HIV described, *"Sleeping without eating happens once in a while, [it] is normal since we are humans. We can sleep without eating, but in the morning, we eat as usual."*

Under difficult economic situations, many participants described relying on family support networks to buffer food insecurity and ensure that PLHIV could access nutritious and preferred foods. As a 22-year-old man who was diagnosed with HIV at age 11 explained: *“My aunt supports me, she sends me some money.”* A woman in her mid-forties diagnosed two years ago explained that “*Sometimes when I do not have money, I just tell my son-in-law and he just sends 30,000 [Tanzanian shillings] for my expenses.”* Being able to rely on family networks for supports helps to ensure that PLHIV can eat.

As a last resort, participants also described purchasing on credit or getting loans; this was the least preferred solution. A 50-year-old PLHIV man explained, *“Taking a loan is not good. It is better to minimize the budget, even [if] only children eat, and you adults sleep [on an] empty stomach, and then you can think on what to do [the] next day but taking a loan ends in shame and humiliation.”* Others, including a 27-year-old caregiver for her HIV+ mother, purchased food on credit from a shopkeeper. She explained, *"You know we are in the environment where people know us; even [if] I do not have money, I can go to a shop and take food. I know that I will work hard if I get money, I pay back [the shopkeeper], then life goes on."*

We found that family food allocations were driven by clinical instructions of which foods to eat so other community members would be unaware of the PLHIVs disease status for fear of stigmatization. Economic challenges impact food affordability. Because the sample population could not afford to cook in separate pots, affecting their ability to consume preferences and/or to eat adequately, the family, who act as the PLHIV’s support network, serves as a structural boundary to PLHIV food choices being met. For this, PLHIV food consumption is highly contingent on what the entire family can afford and prefers to eat. Within this environment, individuals have no choice but to accept the available foods, as a 52-year-old female, who has been HIV+ for three years, mentioned, *"We just cook what is available in the city."* Affordability issues came from living in an economy with an increased cost of living and constant food price fluctuations. Gender plays a role in food organoleptic preferences and how people think about food and the foods they perceive as healthy. We found PLHIV men were satisfied with staple foods, such as porridge, corresponding with their perception of meat, porridge, and fruits as healthy. More aligned with healthcare workers instructions, PLHIV women indicated that a healthy diet and satiety came from consuming a wider variety of foods, such as vegetables, and to reduce meat consumption.

When family members were aware of PLHIV preferences, they enabled PLHIV’s diets whenever possible. Based on their perception of previous experiences of more severe disease states, family members ensured that PLHIV had adequate food for medication adherence to allow for a reduced caregiving burden, feelings of guilt due to PLHIV’s status, or stigma for PLHIV appearing diseased. The family’s motivations facilitate their influence on ensuring that PLHIV preferences are met, connecting the individual to the family food environment, and revealing food choice as a means of social support.

## Discussion

### Drivers of food choice and family food environment among PLHIV families

**Fig. 2:**
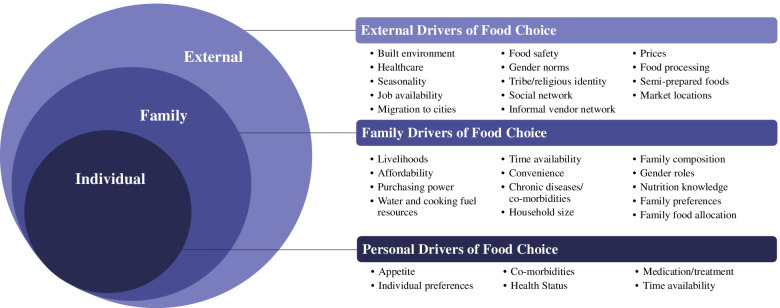
Drivers of food choice among PLHIV in Peri-Urban Dar es Salaam, Tanzania. Based on Giddens [19] and Turner’s [20] frameworks in their influence on PLHIV is based on structure and food choice at the personal, family, and external food environment

As a family, people often buy food, eat together, and share dietary practices. The family food environment is an important intermediary between external and personal food environments. When an individual receives the diagnosis of a chronic disease like HIV and is recommended to make dietary changes, the family food environment plays a key role in enabling optimal food choice and ultimately diets for PLHIV and for the entire family. Here, we examined food choices within various food environment levels (personal and external) and explored how a family influences and strategizes to deal with challenges of the food environment (e.g., prices, affordability, multiple preferences within a family, co-morbidities) using Giddens’ structuration theory and Turner’s food environment framework. Giddens’ structuration theory explains the ways that choices are bounded. When applied to the food environment, prices and availability limit food choices of the PLHIV and family members in peri-urban Tanzanian PLHIV families [21]. Stigma, affordability, and gender were significant structural factors that constrained individual food choices. Figure 2 summarizes the conceptual factors at the external, family, and personal level that structures food choice among PLHIV.

Food holds social values and knowledge of families’ food choice dynamics is vital to limit HIV disease progression and poor health outcomes for PLHIV, their caregivers, and families. For PLHIV, a pattern emerged in which food preferences changed over time since diagnosis. PLHIV and their family members expressed that duration on ARVs caused a change in PLHIV organoleptic food preferences which were amplified when they felt sicker. Food choices reflected decisions made to support the health of both the PLHIV and the whole household. Of course, the economic situation constrained choices and led to creative strategies to meet needs. Family members accommodated PLHIV and limited their own choices to ensure the PLHIV had adequate and desired foods so that they could take their medications. It is important to note that these results reflect the experiences of a sub-population of PLHIV who had disclosed their HIV status to their families and were from one peri-urban community outside of a major urban center in Tanzania. Their personal food choices were influenced by organoleptic, cultural, and emotional preferences. These preferences changed with increased severity of HIV, duration on ARVs, and when co-morbidities arose. Using Giddens' structuration theory as a lens, our data also show that individual agency was constrained by economic challenges, stigma, and gender. Some PLHIV food choices were managed at the family-level while still reflecting individual and external food environment constraints.

These interviews with PLHIV and caregivers allowed us to identify the family as a third domain connecting the external and personal domains. A systemic mapping on low-to-middle-income countries food choices explained the sociocultural factors, sensory appeal, health and nutrition perceptions, and socio-demographic factors prevalent in this peri-urban setting [37]. We acknowledge the food environment's structures while being attentive to culture, tradition, and individual agency, we identify decision-making nodes related to food choice. The family is especially important in the context of chronic diseases, particularly with HIV, to reach optimal health outcomes [24].

A qualitative HIV study in Thailand showed that culture motivated food preferences and that PLHIV preferred the appearance, smell, taste, and textures of foods that resembled familiar Thai foods [8]. Similarly, a qualitative study from Ethiopia reported that religious beliefs and values were a significant aspect of PLHIV nutritional care [9]. In our study, only a few participants directly linked ethnicity or religion to preferences. For example, some linked plantains and meats and avoidance of leftovers to social norms. However, disease-related experience could override the desire for highly valued or preferred foods such as meat. When food preferences were expressed, these often related to prevention of digestive complications, which were more common among PLHIV with more severe or longer disease/ARV duration [2]. This qualitative work set the stage for additional questions. Future mixed methods research can address the influence of ethnic and social identities on individual attitudes and preferences. Contrary to research in Malawi [7], but similar to data from Thailand [8], we found that participants associated the medication with reduced appetite and alterations that made food consumption less appealing. PLHIV generally preferred foods they could cook on their own, potentially seeking independence, similar to work that showed PLHIV desire self-sufficiency [38, 39].

PLHIV families in low resource settings experience economic constraints [7, 9]. Even while struggling with food insecurity and poverty, family members would make sacrifices to provide necessary care to PLHIV. Family members would forgo their own food preferences or implement financial strategies to accommodate the preferences of the PLHIV, including food substitutions, buying on credit or with loans, financial gifts from external family members, and skipping meals. Similar to our study findings, many participants reported swapping desired for less desired food and being unable to plan their food purchases due to the market's frequent price changes [7, 9]. Families enabled food choices despite external food environment constraints. Many family members were motivated to follow clinical advice to meet the unique nutritional needs of PLHIV on ARVs. We found that a family's knowledge of what foods a PLHIV should consume significantly influenced the PLHIVs food consumption. Many participants in our study acknowledged reduced ARVs efficacy if the PLHIV skipped meals. Previous work in Malawi also noted that participants reported following health providers' dietary instructions when possible, depending on food availability and affordability [7]. Caregivers in Lesotho emphasized PLHIV food consumption to reduce disease progression [2] and recognized that adequate food would reduce their caregiver burden while meeting familial obligations. They expressed stigma-related fears related to death or weight changes associated with HIV status and disease severity, which motivated them to support the health of the PLHIV.

Factors influencing PLHIV food choices at the household level interact with family members’ gender, knowledge of healthy foods, and family meal structuring. Research conducted in Zambia, Uganda, India, and Tanzania showed that women are often the preferred at-home caregivers [13, 40–42]. In our sample, female caregivers expressed more knowledge about PLHIV preferences compared to the male caregivers. In part, men might be less cognizant of PLHIV food preferences because men typically do not participate in food preparation or cooking [43].

The disclosure status of PLHIV limits the study findings, i.e., lack of comparability on how family food environments affect PLHIV food choices when their status has not been disclosed. However, a major strength of this study is the exploration of both PLHIV food choice and family member food choices to understand family food environment dynamics (given disclosure and family support). These complementary perspectives allowed us to expand Turner’s food environment framework by adding nuances about how relationships and the nutritional knowledge of other household members impacts food choices when a PLHIV discloses their status to one or more household members.

Previous studies reported that misinformation about HIV transmission meant that family members did not share foods/meals with a PLHIV [13, 44–48]. Our study shows that disclosure of HIV status and accurate knowledge about transmission and nutrition are critical to dispelling such myths. In addition, a holistic understanding of the complexity of food choices is critical to making impactful recommendations to improve nutrition and health [17]. Our expanded framework acknowledges the importance of other household members and the impact of their constraints and strategies to optimize food choice.

## Conclusions

The family perspective is essential for tailoring future HIV nutrition interventions and can improve diets in sustainable and cost-effective ways when food choices and preferences can be nudged towards nutritious foods. In addition, as non-communicable and chronic diseases continue to rise, family-level interventions might improve the health and well-being of PLHIV and positively impact all family members.

## Data Availability

De-identifiable data available upon IRB approval and investigator review. Please contact rambikap@purdue.edu for further information.

## Abbreviations

ARV: Antiretroviral
DECIDE: Diet, Environment, and Choices of positive living
DUCS: Dar es Salaam Urban Cohort Study
HIV: Human immunodeficiency virus
PLHIV: People living with human immunodeficiency virus
Tshs: Tanzanian shillings
